# Variable Effects of PD-Risk Associated SNPs and Variants in Parkinsonism-Associated Genes on Disease Phenotype in a Community-Based Cohort

**DOI:** 10.3389/fneur.2021.662278

**Published:** 2021-04-14

**Authors:** Katerina Markopoulou, Bruce A. Chase, Ashvini P. Premkumar, Bernadette Schoneburg, Ninith Kartha, Jun Wei, Hongjie Yu, Alexander Epshteyn, Lisette Garduno, Anna Pham, Rosa Vazquez, Roberta Frigerio, Demetrius Maraganore

**Affiliations:** ^1^Department of Neurology, NorthShore University HealthSystem, Evanston, IL, United States; ^2^Health Information Technology, NorthShore University HealthSystem, Evanston, IL, United States; ^3^Program for Personalized Cancer Care, NorthShore University HealthSystem, Evanston, IL, United States; ^4^Department of Neurology, Tulane University, New Orleans, LA, United States

**Keywords:** phenotype, genetic association, protein interaction network, gene level tests, community cohort, Parkinson's disease

## Abstract

Genetic risk factors for Parkinson's disease (PD) risk and progression have been identified from genome-wide association studies (GWAS), as well as studies of familial forms of PD, implicating common variants at more than 90 loci and pathogenic or likely pathogenic variants at 16 loci. With the goal of understanding whether genetic variants at these PD-risk loci/genes differentially contribute to individual clinical phenotypic characteristics of PD, we used structured clinical documentation tools within the electronic medical record in an effort to provide a standardized and detailed clinical phenotypic characterization at the point of care in a cohort of 856 PD patients. We analyzed common SNPs identified in previous GWAS studies, as well as low-frequency and rare variants at parkinsonism-associated genes in the MDSgene database for their association with individual clinical characteristics and test scores at baseline assessment in our community-based PD patient cohort: age at onset, disease duration, Unified Parkinson's Disease Rating Scale I-VI, cognitive status, initial and baseline motor and non-motor symptoms, complications of levodopa therapy, comorbidities and family history of neurological disease with one or more than one affected family members. We find that in most cases an individual common PD-risk SNP identified in GWAS is associated with only a single clinical feature or test score, while gene-level tests assessing low-frequency and rare variants reveal genes associated in either a unique or partially overlapping manner with the different clinical features and test scores. Protein-protein interaction network analysis of the identified genes reveals that while some of these genes are members of already identified protein networks others are not. These findings indicate that genetic risk factors for PD differentially affect the phenotypic presentation and that genes associated with PD risk are also differentially associated with individual disease phenotypic characteristics at baseline. These findings raise the intriguing possibility that different SNPs/gene effects impact discrete phenotypic characteristics. Furthermore, they support the hypothesis that different gene and protein-protein interaction networks that underlie PD risk, the PD phenotype, and the neurodegenerative process leading to the disease phenotype, and point to the significance of the genetic background on disease phenotype.

## Introduction

Parkinson's disease (PD), the second most common neurodegenerative disease, has an insidious onset and a long pre-symptomatic and symptomatic course. Four cardinal features that include resting tremor, bradykinesia, rigidity, and postural instability define the motor aspects of the disease. The constellation of clinical symptoms however is variable both in terms of symptom combination and temporal profile. This variability has led to phenotypic classification according to different disease characteristics. A commonly accepted classification is based on motor symptoms: disease subtypes include a tremor-predominant, akinetic/rigid, and mixed subtype (1). More recently, additional classifications have emerged based on different clinical features such as non-motor features, disease progression, a combination of motor and non-motor features, combination of clinical features and comorbidities, multimodal imaging and genetic burden. More specifically, Sauerbier et al. (2) in their review proposed the existence of a distinct non-motor subtype (NMS) of NMS-dominant PD based on the burden of non-motor symptoms in early PD including cognitive dysfunction, anosmia, anxiety, depression, sleep disorders, and autonomic dysfunction observed either alone or in varying combinations. Simuni et al. (3) reported that, for the Primary Progression Markers Initiative (PPMI) PD cohort, higher baseline non-motor scores were associated with female sex and a more severe motor phenotype. Longitudinal increase in non-motor score severity was associated with older age and lower CSF aβ1–42 at baseline. Lawton et al. (4) identified four phenotypic clusters in their cohort: (1) fast motor progression, (2) mild motor and non-motor disease, (3) severe motor disease, poor psychological well-being and poor sleep with intermediate motor progression, and (4) slow motor progression with tremor-dominant unilateral disease. Mollenhauer et al. (5) in their analysis of the *De Novo* Parkinson (DeNOPA) cohort, reported that baseline predictors of worse progression of motor symptoms included male sex, orthostatic blood pressure drop, diagnosis of coronary artery disease, arterial hypertension, elevated serum uric acid, and CSF neurofilament light chain.

A variable temporal profile of motor symptom appearance and progression has been reported in different cohorts that have been followed longitudinally for different lengths of time and identified predictors of disease progression and phenotypic clusters. In the DeNOPA cohort, predictors of cognitive decline in PD included previous heavy alcohol abuse, current diagnoses of diabetes mellitus, arterial hypertension, elevated periodic limb movement index during sleep, decreased hippocampal volume by MRI, and higher baseline levels of uric acid, C-reactive protein, high density lipoprotein (HDL) cholesterol, and glucose. In their cohort, risk markers for faster disease progression included cardiovascular risk factors, deregulated blood glucose, uric acid metabolism and inflammation. In the PPMI cohort, Aleksovski et al. (6) reported that the postural instability gait disorder (PIGD) subtype, compared to the tremor-predominant subtype, was characterized by more severe disease manifestations at diagnosis, greater cognitive progression, and more frequent psychosis (5). In the PPMI cohort, Latourelle et al. (7) found that higher baseline MDS-UPDRS motor score, male sex, and increased age, as well as a novel Parkinson's disease-specific epistatic interaction, were indicative of faster motor progression. In their retrospective review of a cohort of 100 autopsy confirmed PD cases, Pablo-Fernandez at al. (8) reported that the presence of autonomic dysfunction defined as autonomic failure on autonomic testing or the presence of at least two symptoms such as urinary symptoms, constipation, orthostatic hypotension, or sweating abnormalities was associated with a more rapid progression and shorter survival.

Other classifications of disease subtypes have been proposed in addition to motor, non-motor symptom and disease course-based classifications. Inguanzo et al. (9) employed a radiomics and hybrid machine learning approach to identify mild, intermediate and severe disease subtypes based on a combination of dopaminergic deficit by imaging and escalating motor and non-motor manifestations.

In the last two decades, genome-wide association studies (GWAS) of common genetic variants and dissection of the low frequency and rare variants contributing to familial forms of PD has implicated an increasing number of genetic loci in disease risk and severity. This has cemented the view that PD is a complex and heterogeneous genetic disorder, with variants at many genes impacting disease phenotype and course. We are just beginning to understand whether PD-risk variants are differentially associated with baseline features or disease subtype. Tan et al. (10) performed a GWAS of motor and cognitive progression in PD and reported that *ATPBB2*, a phospholipid transporter related to vesicle formation, is associated with motor progression, and that variants at *APOE* drive cognitive progression, whereas there was no overlap of variants associated with PD risk and PD age-at-onset with disease progression. Iwaki et al. (11) demonstrated sex-specific SNP associations with features of the PD phenotype: female patients had a higher risk of developing dyskinesias and a lower risk of developing cognitive impairment. Periñán et al. (12) reported an association of the TT genotype at the *PICALM* SNP rs3851179 with a decreased risk of cognitive impairment in PD. *GBA* variants have been associated with PD and generally are associated with faster progression and more severe phenotypes (13, 14). Blauwendraat et al. (15) reported that in a large PD patient cohort, *GBA* risk variants decrease age at onset in PD.

Genetic factors that increase the risk of PD and genetic factors that affect disease severity and progression are not necessarily identical. Furthermore, individual genetic factors that influence disease severity and progression may not have an immediately identifiable impact in the clinical practice setting. It is therefore important to consider the predictive ability and significance of the impact of genetic variation on individual phenotypic characteristics and parameters that are clinically relevant and may have treatment implications (16–18). If one or a set of genetic variants contribute differentially to a particular phenotypic characteristic, it will be challenging to discover them using GWAS or gene-level association tests in a genome-wide screen since phenotypically well-characterized cohorts are typically modest in size, making it unlikely to discover genome-wide significant associations. We have therefore taken a focused approach, choosing to evaluate possible associations with SNPs that have been previously demonstrated to show significant associations with PD using large GWAS and low frequency and rare variants at parkinsonism-associated genes identified in the MDSgene database (19), hypothesizing that these genetic variants may differentially contribute to baseline clinical parameters/symptoms. Under this hypothesis, evaluating their association in a smaller cohort of subjects where individual clinical symptoms and objective test scores are obtained at baseline using structured clinical documentation support (SCDS) tools embedded in the electronic medical record (EMR) in a routine clinical practice setting (20) could allow for the discovery of significant associations. This would not be possible in the context of a case-control GWAS.

Indeed, we find that common SNPs from PD-risk genes identified in GWAS are individually associated with a range of clinical features: family history of dementia, the presence of hallucinations, bradykinesia, depression, orthostatism, disease subtype, and complications of levodopa therapy. When low-frequency and rare variants at PD-risk genes and parkinsonism-associated genes are analyzed in gene-level tests, associations with clinical characteristics such as presence of bradykinesia, depression, autonomic symptoms (orthostatism, constipation) UPDRS motor scores, mentation, complications of therapy scores, H&Y stage, and a family history of dementia are identified. All of the associations we report survive Bonferroni correction and some approach or reach genome-wide significance. It is interesting to note that the gene associations identified from the analysis of individual common SNPs do not always overlap with those identified in gene-level tests using low-frequency and rare variants suggesting an important role of the genetic background on the phenotypic manifestations.

## Methods

### Subjects and Clinical Information

Eight hundred and fifty-six subjects with clinically definite or clinically probably Parkinson's disease (Bower criteria) (21) enrolled in two previously described patient cohorts [Molecular Epidemiology of Parkinson's Disease, MEPD (22), *N* = 201; DodoNA (23), *N* = 655] were included in this study. All patients in these cohorts had a diagnosis of PD at study entry and were residents of Cook and Lake Counties in Illinois, USA. Though both cohorts include individuals with diverse ancestries, the filtering described in the following section restricted the analysis to 786 individuals of European ancestry: 504 males, 282 females. Blood samples were collected in the majority of cases at an initial baseline visit or within a 3-month window following the initial visit. Data on clinical parameters were obtained from SCDS developed to standardize clinical assessment and retained within the EMR as described (20, 23). Given the community-based practice setting, our cohort included both *de novo* and previously diagnosed PD patients.

The following phenotypic characteristics were analyzed in our cohort: initial motor and non-motor symptoms as reported by the patient, as well as motor and non-motor symptoms identified by the clinician at their baseline encounter. Objective clinical assessment at the baseline encounter included scores on the Mini-mental Status Evaluation (MMSE) / Montreal Cognitive Assessment (MoCA) or Short Test of Mental Status (STMS) (24–26). Due to copyright limitations, cognitive status was assessed initially using the MMSE, at a later time-point the MoCA, and finally the STMS. The individual test scores on the MoCA and STMS were converted to MMSE scores using established normograms prior to analysis (26, 27). Objective clinical assessments at the baseline encounter also included scores on the Unified Parkinson's Disease Rating Scale (UPDRS) (28) [I – Mentation, Behavior and Mood; II – Activities of Daily Living; III – Motor Examination; IV – Complications of Therapy; V – Hoehn &Yahr stage; VI – Schwab & England Activities of Daily Living Scale], Epworth sleepiness scale (ESS) (29) and Geriatric Depression scale (GDS) (30), information on family history of PD, dementia, stroke, epilepsy, multiple sclerosis, and neuropathy, as well as information on comorbidities including diabetes, cardiovascular disease, migraine, schizophrenia, anxiety, depression, peripheral neuropathy and sleep apnea. Supplementary Table 1 presents the list of clinical parameters and descriptive statistics for these parameters. Treatment details including medical and surgical therapy were collected but not included in the analysis presented here.

### Genotyping and Quality Control Measures

Blood samples were stored at −80°C until DNA was extracted. Genotypes were obtained by interrogating an Affymetrix Axiom™ genome-wide human array containing 531,674 variants that included custom content, specifically variants at genes associated with PD and other neurological disorders. Prior to imputation using IMPUTE2 (31) against the 1,000 Genomes Phase 3 CEU genome, subjects were filtered in PLINK 1.07 (32) or 1.9 (33) for low overall genotyping rates (<95%) and sex-discordance, and variants with >5% missing calls were removed from the analysis. Imputed SNPs were retained only if *R*^2^ ≥ 0.90. Only subjects with European ancestry were retained by using principal components one and two (PC1 and PC2) from a principal components analysis (PCA) with 103 ancestry informative markers (AIMs). For association tests with single variants, variants were also filtered by Hardy-Weinberg test statistic (1 × 10^−4^) and to have a minor allele frequency (MAF) > 1%.

### Association Tests

Genes and variants initially identified for testing association with clinical parameters were selected based on a prior demonstrated association with PD/parkinsonism or disease progression [MDSgene.org; (10–12, 34–38)], or because the gene harbors pathogenic variants that cause PD/parkinsonism [for review, see (39, 40)]. Of 168 variants with a previously reported association and a MAF > 1%, 138 variants (Supplementary Table 2) were present in our data after filtering as described above. These were tested using PLINK for association applying logistic regression for binomial variables if at least 3% of subjects (*N* = 24) displayed the clinical parameter, or linear regression with standardized (mean 0, standard deviation 1) scaled variables and reverse scoring the MMSE so that worse scores indicate poorer performance. Associations were evaluated for both sexes jointly and for each sex separately. Sex, age-at-encounter and, since our community-based cohort includes both *de novo* and previously diagnosed patients, years-from-diagnosis were included as covariates for associations evaluated in both sexes, age-at-encounter, and years-from-diagnosis as covariates for associations evaluated in just one sex, and years-of-education added as an additional covariate for tests of association with cognitive measures (MMSE).

After using PLINK 1.9 to convert binary files to a VCF format, CHECKVCF (https://github.com/zhanxw/checkVCF) was used to verify the quality of the VCF file and TABANNO (https://github.com/zhanxw/anno) was used to annotate genes relative to NCBI build 37 (hg19). Gene-level association testing performed was using the sequence kernel association test (SKAT) (41) as implemented in RVTESTS (42), with and without the covariates: sex, age-at-encounter, years-from-diagnosis, and for MMSE, years of education (https://github.com/zhanxw/rvtests), using default parameters [significance evaluated using 10,000 permutations at alpha = 0.05, weight = Beta (beta1 = 1.00, beta2 = 25.00), missing genotypes imputed to mean], variant filtering to include non-synonymous, start-gain, stop-gain, stop-loss, start-loss, frameshift, codon-gain, codon-loss, codon-region, insertion, deletion, essential-splice-site, normal-splice-site, and structural-variation variants, filtering to include both rare (<1% MAF) and low-frequency (1–5% MAF) variants or only rare variants, and including only genes with at least two variants (*N* = 117 for MAF ≤ 5%, *N* = 106 for MAF ≤ 1%, Supplementary Table 3). An association was considered significant if the Bonferroni-corrected *p-*value was < 0.05.

### Protein-Protein Interaction Network Evaluation

To evaluate whether the genes whose variants exhibited significant associations with clinical parameters identify protein products that are members of a functional protein-protein interaction network, those genes were entered into the Search Tool for the Retrieval of Interacting Genes, STRING, v.11 (43).

## Results

We hypothesized that SNPs which have been previously demonstrated to show significant associations with PD-risk using large GWAS and low frequency and rare variants at parkinsonism-associated genes identified in the MDSgene database (19) differentially contribute to discrete baseline clinical parameters/symptoms. To test this hypothesis, we evaluated their association in two well-characterized patient cohorts [MEPD (20) and DodoNA (23)] where individual clinical symptoms and objective test scores were obtained at baseline using SCDS tools embedded in the EMR. The findings are presented in the following two sections.

### Single SNP Association Analyses

We initially evaluated whether common SNPs that have been previously associated with PD-risk in large GWAS are also associated with distinct binomial clinical phenotypic features of PD at their baseline presentation. We find significant associations that are at times sex-specific, and that the significant SNPs are typically located in non-overlapping genes/regions (Table 1).

**Table 1:** Associations of binomial traits with individual common PD-risk SNPs^*^.

**Clinical feature**	**SNP**	**Nearest gene**	**Distance (kb)**	**MAF this study**	**Sex**	**Model**	**unadjusted *p*-value**	**Bonferroni corrected *p*-value**	**OR**	**95% CI**
Family history of dementia *n* = 1	rs2280104 rs10253857	*BIN3* *SNX13*	0 116.9	33.0 23.6	Males Both	GENO-2DF GENO-2DF	1.64 × 10^−4^ 3.55 × 10^−4^	0.0186 0.0415	NA NA	
Family history of dementia *n* ≥ 2	rs429358 APOE ε4 dose	*APOE*	0	12.4 11.1	Females Females Females Females	Additive GENO-2DF Additive GENO-2DF	7.36 × 10^−5^ 3.86 × 10^−4^ 7.26 × 10^−5^ 4.75 × 10^−4^	0.00375 0.0197 0.00370 0.0242	8.92 NA 7.42 NA	3.02–26.3 2.72–20.3
Family history of dementia *n* ≥ 1	rs2280104	*BIN3*	0	33.0	Males Males	Additive GENO-2DF	2.91 × 10^−4^ 8.95 × 10^−5^	0.0340 0.0105	1.76 NA	1.30–2.39
Family history of stroke *n* = 1	rs2074404	*WNT3*	0	4.07	Males	GENO-2DF	1.28 × 10^−6^	1.42 × 10^−4^	NA	
Presence of neuropathy	rs1867598 rs2694528	*ELOVL7* *NDUFAF2*	0 0	13.5 13.4	Both Both Females Both Both	Additive GENO-2DF Additive Additive GENO-2DF	3.02 × 10^−5^ 1.33 × 10^−4^ 3.00 × 10^−4^ 2.97 × 10^−5^ 1.30 × 10^−4^	0.00296 0.0130 0.0242 0.00291 0.0130	3.8 NA 5.92 3.8 NA	2.04–7.18 2.26–15.5 2.04–7.19
History of essential tremor	rs12528068	*RIMS1*	108.6	13.5	Males	Additive	4.51 × 10^−4^	0.0491	2.06	1.38–3.09
**Initial motor symptoms**										
Bradykinesia – any	rs8192591	*NOTCH4*	0	3.18	Both	GENO-2DF	1.71 × 10^−5^	0.00206	NA	
Bradykinesia – reduced dexterity	rs1293298	*CTSB*	0	26.8	Both	GENO-2DF	5.20 × 10^−4^	0.0499	NA	
Bradykinesia – reduced arm swing	rs34311866	*TMEM175*	0.5	25.3	Both	Additive	1.82 × 10^−4^	0.0160	2.31	1.50–3.57
**Initial non-motor symptoms**										
Depression	rs61169879	*BRIP1*	0	14.9	Males	Additive	2.84 × 10^−4^	0.0304	2.60	1.55–4.37
**Non-motor symptoms at baseline**										
Hallucinations	rs55961674	*KPNA1*	0	19.9	Males	Additive	1.72 × 10^−4^	0.0161	2.84	1.65–4.90
Insomnia	rs117615688	*CRHR1*	0	5.92	Females	GENO-2DF	1.87 × 10^−4^	0.0179	NA	
Restless leg syndrome	rs382940	*SLC44A1*	0	6.79	Both	GENO-2DF	5.14 × 10^−4^	0.0483	NA	
Orthostatism	rs34025766	*LCORL*	0	17.0	Males	Additive	2.14 × 10^−4^	0.0218	2.91	1.65–5.12
**Disease subtype**										
Tremor-predominant subtype	rs9468199^**^	*LOC100507172*	3.2	17.4	Both	Additive	5.55 × 10^−4^	0.0765	2.10	1.37–3.20

* *Covariates: sex, age-at-encounter, years-since-diagnosis, and for MMSE, years of education*.

** *Fails to sustain significance if years-from-diagnosis is included as a covariate*.

Using an additive model, female PD patients carrying the minor allele (T) at SNP rs429358 at *APOE*, or having an *APOE* ε4 allele have an ~8-fold increased risk of having a positive family history of more than one family member with dementia. Individuals with the minor allele (T) at SNP rs3431186 at *TMEM175*, which encodes a potassium channel that regulates lysosomal membrane potential and pH stability in neurons (44), are about twice as likely to have reduced arm swing, a manifestation of bradykinesia. Male PD patients with the minor allele (T) at SNP rs5396167 in *KPNA1*, which encodes importin α5 and is involved in lysosomal biogenesis and autophagy (45, 46), have a 2.8-fold increased risk to have hallucinations at baseline. Males with the minor (T) allele at SNP rs12528068 108.6 kb from the *RIMS1* gene, which encodes one of four isoforms of presynaptic scaffolding proteins involved in synaptic transmission (47), have a 2.1-fold increased risk of a history of essential tremor. Individuals carrying the minor allele (G) at SNP rs186798 in *ELOVL7* have a 3.8-fold increased risk to also have a prior diagnosis of peripheral neuropathy. The *ELOVL7* gene is a PD risk factor that also confers regional vulnerability, i.e., it is a Braak stage-related gene with an altered expression pattern in the brains of PD cases, with down regulated expression in endothelial cells and oligodendrocytes (48) (Table 1).

Additional associations are identified using the GENO-2DF model, which considers both additive and dominance effects. SNP rs2280194 in *BIN3* and rs10253857 in an intergenic region near *SNX13* are associated with a family history of dementia. SNP rs2074404 in *WNT3* is associated with a family history of stroke. SNP rs2694528 in *NDUFAF2*, which is near *ELOVL7*, is associated with the presence of neuropathy. SNPs rs8192591 in *NOTCH4* and rs1293298 in *CTSB* are associated with bradykinesia as an initial motor symptom. SNPs rs117615688 in *CRHR1* and rs382940 in *SLC44A1* are associated the non-motor symptoms of insomnia and restless leg syndrome (RLS), respectively (Table 1).

We also identified significant associations between common SNPs conferring risk of PD in GWAS and test scores that reflect an objective assessment of the PD patient (Table 2). The minor allele (T) at SNP rs12528068 in an intergenic region 108.6 kb from *RIMS1* that is associated with a history of essential tremor in males is also associated with increased dyskinesia scores in females. SNPs rs113343 and rs6497339 at *SYT17*, which encodes synaptotagmin-17, are associated with higher GDS scores. SNP rs12283611 at *DLG2*, which functions in the clustering of receptors, ion channels and associated signaling proteins, is associated with lower UPDRS-VI scores.

**Table 2:** Significant associations of test scores with common PD-risk SNPs^*^.

**Clinical feature**	***SNP***	**Nearest gene**	**Distance to nearest gene (kb)**	**MAF this study**	**Sex**	**Model**	**Unadjusted *p*-value**	**Bonferroni corrected *p*-value**	**β**	**95% CI**
GDS score	rs11343 rs6497339	*SYT17* *SYT17*	0 0	43.1 43.8	Males Both Both	GENO-2DF GENO-2DF GENO-2DF	1.35 × 10^−4^ 7.17 × 10^−5^ 2.22 × 10^−4^	0.0166 0.00910 0.0281	NA NA NA	
UPDRS IV- dyskinesia subscore	rs12528068	*RIMS1*	108.6	28.6	Females	Additive GENO-2DF	5.25 × 10^−5^ 1.33 × 10^−4^	0.00635 0.0161	0.254 NA	0.132–0.375
UPDRS VI-Schwab and England	rs12283611	*DLG2*	0	44.0	Males	GENO-2DF	1.98 × 10^−4^	0.0243	NA	

* *Covariates: sex, age-at-encounter, years-since-diagnosis, and for MMSE, years of education*.

We included years-from-diagnosis as a covariate in the above analyses since our community-based cohort includes previously diagnosed patients. It is interesting that some results that trended toward significance survive Bonferonni correction if this measure of disease duration is not included as a covariate (Supplementary Tables 4, 5). Individuals with the minor (A) allele at the SNP rs9468199 in an intergenic region 3.2 kilobases (kb) from *LOC1005071*, an uncharacterized non-coding RNA, are twice more likely to present with the tremor-predominant PD subtype and not the akinetic/rigid or mixed disease subtype. The minor (C) allele at SNP rs12813102 in *GPR19*, which encodes a proton-sensing G-protein coupled receptor abundant in skin and brain (49), has a relatively strong effect on higher H&Y stage (β ~1.7 on standardized H&Y scores) in both sexes or just males.

In contrast, other SNPs have less strong effect sizes (β range 0.24–0.47 on standardized scores). The presence of the minor allele (C) at SNP rs823118 in *NUCKS1*, which is involved in homologous recombination DNA repair (50), is associated with higher MMSE baseline scores only in males. Its small effect is not unexpected given that early in the disease process, cognitive impairment is not prominent in typical PD. Finally, the minor allele (T) at SNP rs224750 located 167.5 kb from *PARD3* is associated with higher UPDRS-IVc scores only in females. *PARD3* is a gene involved in the regulation of cellular junction formation in ependymal cells, cilia, tumor suppression (49). It will be useful to evaluate these variants in longitudinal follow-up studies.

In summary, these results collectively demonstrate that some of the PD-risk SNPs identified in case-control GWAS are also associated with the differential presentation of PD and discrete phenotypic characteristics at baseline.

### Gene-Level Association Analysis

We employed gene-level association tests (sequence kernel association tests) to evaluate whether the set of rare (MAF < 1%) or both rare and less common (MAF < 5%) variants present in the PD-associated genes of our cohorts also exert differential effects on baseline clinical features. Significant findings from these gene-level association tests in our cohorts are presented in Table 3. The following findings are notable: *LRRK2* is associated with a prior diagnosis of essential tremor (ET). *NUCKS1*, a gene that shows allele-specific gene expression in the human brain (51), is significantly associated with UPDRS-III motor scores and with UPDRS-V (H&Y stage). Of note, the PD-risk SNP rs823118 in the same gene was associated with higher MMSE scores in males when disease duration was not included as a covariate (Supplementary Table 5).

**Table 3:** Significant associations in gene-based sequence kernel association tests^*^.

**Clinical feature**	**Analysis *N***	***N* with condition in this study (%)**	**Variant MAF criteria**	**Gene**	**Number of variants**	**Unadjusted *p* (permutation *p*)**	**Bonferroni corrected *p***
**Clinical history**							
Essential tremor	779	144 (18.3)	≤ 5%	*LRRK2*	37	3.93 × 10^−5^ (0)	0.00460
**Test scores**							
MMSE	783	-	≤ 1%	*FAM171A1*	15	2.99 × 10^−4^ (0.0102)	0.0317
UPDRS III	743	-	≤ 1%	*NUCKS1*	8	3.87 × 10^−4^ (0.0012)	0.0410
UPDRS -I	768	-	≤ 1%	*TMEM163*	19	3.33 × 10^−4^ (0.0012)	0.0353
UPDRS IV-total score	786	-	≤ 1% ≤ 1%	*TOX3* *SULT1C2*	16 6	3.55 × 10^−5^ (0.0014) 3.59 × 10^−4^ (0.0036)	0.00376 0.0381
UPDRS-V-H&Y stage	773	-	≤ 1% ≤ 5%	*NUCKS1* *NUCKS1*	8 11	1.34 × 10^−4^ (0.0003) 1.90 × 10^−4^ (0.0002)	0.0223 0.0142
UPDRS-VI-Schwab & England	773	-	≤ 1%	*TRIM40*	2	1.22 × 10^−6^ (0.0004)	0.000130
Dyskinesia at baseline: chorea	675	61 (7.7)	≤ 5%	*FAM184A*	60	3.55 × 10^−4^ (0.0005)	0.0416
Dyskinesia severity at baseline: severe	784	61 (7.7)	≤ 5%	*FAM184A*	60	1.71 × 10^−4^ (0.0012)	0.00201
Dyskinesia distribution at baseline: generalized	786	30 (3.8)	≤ 1% ≤ 5%	*SNCA* *FAM184A*	7 60	2.17 × 10^−4^ (0.0351) 1.29 × 10^−5^ (0.0003)	0.0230 0.00151
**Initial motor symptoms**							
Bradykinesia (reduced dexterity)	786	40 (5.1))	≤ 1%	*HLA-DQB1*	1	4.40 × 10^−4^ (0.0108)	0.0466
Bradykinesia (generalized)	786	65 (8.2)	≤ 1%	*SCAF11*	15	4.43 × 10^−4^ (0.0149)	0.0469
Bradykinesia (micrographia)	786	30 (3.8)	≤ 1%	*CHD9*	30	2.40 × 10^−5^ (0.0037)	0.00254
Postural tremor	786	140 (17.8)	≤ 1%	*FAM49B*	15	3.62 × 10^−5^ (0.0005)	0.00389
Rigidity	786	681 (86.4)	≤ 5%	*GPNMB*	20	2.26 × 10^−4^ (0.0082)	0.0265
**Motor symptoms at baseline**							
Bradykinesia	786	779 (98.9)	≤ 1% ≤ 5% ≤ 1% ≤ 1%	*GPNMB* *GPNMB* *MCCC1* *THSD4*	17 20 14 133	1.18 × 10^−8^ (0.0072) 5.48 × 10^−5^ (0.0135) 6.61 × 10^−5^ (0.0180) 3.08 × 10^−4^ (0.0066)	1.25 × 10^−6^ 0.00641 0.00700 0.0361
**Initial non-motor symptom**							
Depression	786	125 (15.9)	≤ 1%	*ITGA8*	26	2.47 × 10^−4^ (0.0017)	0.0262
**Non-motor symptoms at baseline**							
Cognitive impairment	786	106 (13.5)	≤ 5%	*SNCA*	19	2.90 × 10^−5^ (0.0001)	0.00391
Constipation	786	157 (19.9)	≤ 1%	*ITGA8*	26	1.5 × 10^−5^ (0.0001)	0.00444
Orthostatism	786	56 (7.1)	≤ 5%	*PET117*	4	1.74 × 10^−4^ (0.0217))	0.0238
UPDRS IV-Orthostasis	786	62 (7.9)	≤ 5% ≤ 1%	*STBD1* *STBD1*	2 2	7.40 × 10^−5^ (0.0014) 7.40 × 10^−5^ (0.002)	0.0419 0.00784
Hallucinations	786	49 (6.2)	≤ 5%	*LRRK2*	36	1.68 × 10^−5^ (0.0001)	0.0419
Dysphagia	786	47 (6.0)	≤ 1%	*FAM49B*	15	1.81 × 10^−4^ (0.0052)	0.0192
Anxiety	786	50 (6.3)	≤ 1%	*CATSPER3*	7	1.58 × 10^−7^ (0.0008)	1.68 × 10^−5^
Unexplained weight loss	786	26 (3.3)	≤ 1%	*SQRDL* *RIMS1*	6 72	1.48 × 10^−4^ (0.0091) 4.68 × 10^−5^ (0.0027)	0.01566 0.0496
Restless leg syndrome	786	31 (3.9)	≤ 1%	*STK39*	20	2.24 × 10^−5^ (0.0021)	0.00238
Excess daytime sleepiness	786	60 (7.6)	≤ 1%	*ITGA8*	26	1.83 × 10^−4^ (0.0028)	0.0194
**Family history**							
Dementia (1 family member)	786	160 (20.3)	≤ 1%	*SLC44A1*	16	1.48 × 10^−4^ (0.0009)	0.0156
Dementia (≥ 2 family members)	786	24 (3.0)	≤ 5%	*ASXL3* *LAMB2*	27 5	1.50 × 10^−5^ (0.0044) 4.19 × 10^−4^ (0.0105)	0.00176 0.0490
Dementia (≥ 1 family member)	786	184 (23.4)	≤ 1%	*ASXL3* *SLC44A1*	27 16	5.00 × 10^−5^ (0.0003) 4.47 × 10^−4^ (0.0006)	0.00530 0.0474
Tremor (≥ 2 family members)	786	31 (3.9)	≤ 1%	*GAK*	20	8.70 × 10^−5^ (0.0067)	0.00922
Tremor (≥ 1 family member)	786	121 (15.4)	≤ 1%	*BIN3*	10	1.35 × 10^−4^ (0.0003)	0.014
**Comorbidities**							
Anxiety disorder	786	33 (4.2)	≤ 1%	*BIN3*	10	3.12 × 10^−4^ (0.0057)	0.033
Sleep apnea	786	68 (8.6)	≤ 1%	*VAMP4*	4	3.01 × 10^−4^ (0.0041)	0.0319
Traumatic brain injury	786	41 (5.2)	≤ 5%	PET117	4	6.87 × 10^−7^ (0.0109)	8.03 × 10^−5^

* *Covariates: sex, age-at-encounter, years-since-diagnosis, and for MMSE, years of education*.

*TOX3*, a transcriptional co-activator (52) previously associated with periodic leg movements during sleep (53), and *SULT1C2*, a cytosolic sulfotransferase (54), are associated with the UPDRS-IV total score. *TRIM40*, a gene whose protein product may function as a E3 ubiquitin-protein ligase (55) and inhibit NF-kB activity, is associated with UPDRS-VI (Schwab & England score). *SNCA* and *FAM184A* are associated with dyskinesias at baseline encounter, and *SNCA* is also associated with cognitive impairment. *CHD9*, which encodes a transcriptional activator (56) and *GPNMB*, which encodes a transmembrane glycoprotein (57), are associated with the initial motor symptoms of micrographia (bradykinesia manifestation) and rigidity, respectively. *GPNMB*, demonstrating genome-wide significance, is also associated with bradykinesia at baseline, as are *THSD4*, which attenuates TGFβ signaling, and *MCCC1*, which is used in NFκB signaling (58). *STK39*, which encodes a protein kinase that may mediate stress-activated signals (51), is associated with RLS.

Certain comorbid conditions often seen in PD patients are associated with the different genes. *BIN3*, which encodes a protein involved in cytokinesis (59) is associated with anxiety disorder. *VAMP4* (60) is associated with sleep apnea. *PET117*, which encodes a mitochondrial protein homolog (61), is associated with traumatic brain injury.

Similar results are obtained when sequence kernel association tests are performed without including sex, age at encounter, disease duration and, for MMSE, years of education, as covariates (Supplementary Table 6). In these analyses, different measures of complications of levodopa therapy are associated with some of the genes described above: *TOX3* and *SULT1C2* are associated with the UPDRS-IVa-Dyskinesia subscore; *SULT1C2, MCCC1, TOX3*, and *BAG3* are associated with the UPDRS-IVb-Fluctuations subscore; and *STBD1* is associated with the UPDRS-IVc-Other subscore.

In summary, these results demonstrate that variants in PD-associated genes are differentially associated with the following phenotypic features: history of essential tremor, initial motor and non-motor symptoms, test scores, motor and non-motor symptoms at baseline study entry, family history of essential tremor and of dementia, and comorbidities including anxiety, sleep apnea, and traumatic brain injury (TBI). These associations raise the possibility of underlying links between PD, essential tremor, mood disorders, and TBI.

### Protein-Protein Interaction Network Analysis

**Figure 1:**
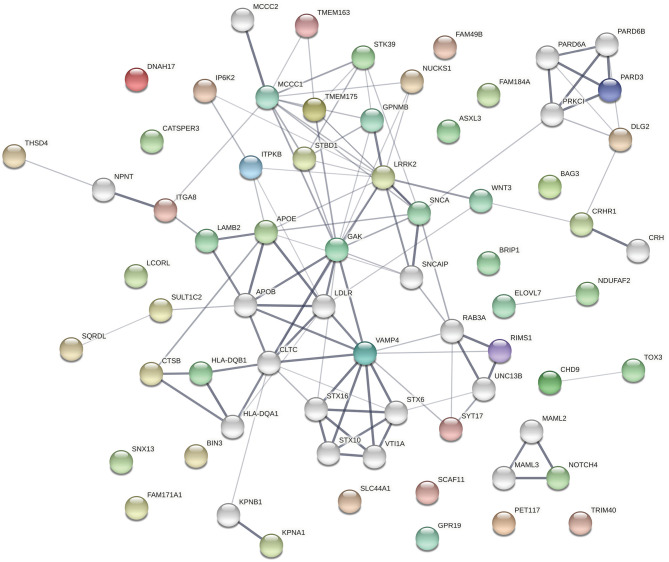
The network shown was obtained using 0.4 as the minimum required interaction score (medium confidence). In the network, the thickness of the lines corresponds to the confidence of the interactions, the colored spheres represent the 49 proteins of the genes identified in the association analyses, and uncolored spheres represent up to 20 second-shell interactions that reveal indirect interactions among the proteins. The network contains 69 nodes and 113 edges (46 expected) with an average node degree of 3.28 and an enrichment *p*-value of 1.11 × 10^−16^. Sixteen nodes are unconnected to the protein-interaction network.

The protein products of the genes included in this analysis s are involved in many different cellular processes implicated in neurodegeneration. To assess whether the significant associations between SNPs/genes with baseline clinical parameters identified here reflect functional interactions between the genes, we entered all of the genes identified as having significant associations with a phenotypic feature (i.e., all genes listed in Tables 1–3, Supplementary Tables 4–6) into the Search Tool for the Retrieval of Interacting Genes (STRING) and evaluated their participation in protein-protein interaction (PPI) networks. The network shown in Figure 1 was obtained using 0.4 as the minimum required interaction score (medium confidence) and allowed up to 20 second-shell interactions to reveal indirect interactions among these proteins. The network contains 69 nodes and 113 edges (*cf*. 46 expected) with an average node degree of 3.28 and an enrichment *p*-value of 1.10 × 10^−16^. Sixteen nodes are unconnected to the protein-interaction network.

The top 30 Gene Ontology (GO) processes in which these genes and their interactors are implicated are shown in Table 4, with the genes having significant SNP or gene-level associations highlighted in bold. It is interesting to note that this analysis reveals three interaction patterns: one in which proteins encoded by genes such as *APOE, KPNA1, LRRK2, TMEM175, MCCC1, FAM49B*, and *SNCA* are members of closely interacting networks, a second one in which genes such as *PARD3* or *NUCKS1* are members of more remotely interacting networks, and a third one in which genes such as *ELOVL7, GPR19, LCORL, FAM184A*, and *BIN3* are not nodes in these protein-interaction networks.

**Table 4:** Top gene ontology enrichment processes in the PPI.

**Gene ontology term description**	**Observed gene count**	**Background gene count**	**Strength**	**False discovery rate**	**Matching proteins in network**
Vesicle fusion	7	95	1.32	8.84 × 10^−5^	**VAMP4**, STX6, **TMEM175**, **SYT17**, STX16, VTI1A, STX10
Cellular component organization	38	5163	0.32	8.84 × 10^−5^	PARD6A, RAB3A, **WNT3**, APOB, **VAMP4**, **APOE**, STX6, **BRIP1**, **TMEM175**, **MCCC1**, **BIN3**, KPNB1, PRKCI, **NDUFAF2**, **LRRK2**, **GAK**, **SNCA**, MCCC2, **KPNA1**, **THSD4**, **SYT17**, **NUCKS1**, **SCAF11**, STX16, PARD6B, **PARD3**, **NOTCH4**, **ITGA8**, UNC13B, VTI1A, **LAMB2**, NPNT, **PET117**, **RIMS1**, LDLR, **CHD9**, STX10, CLTC
Vesicle-mediated transport	22	1699	0.56	8.84 × 10^−5^	RAB3A, APOB, **VAMP4**, **STBD1**, **APOE**, STX6, **TMEM175**, KPNB1, PRKCI, **LRRK2**, **GAK**, **SNCA**, **CTSB**, **SYT17**, STX16, UNC13B, VTI1A, **RIMS1**, **FAM49B**, LDLR, STX10, CLTC
Regulation of localization	26	2524	0.47	8.84 × 10^−5^	PARD6A, RAB3A, **WNT3**, APOB, **APOE**, STX6, SNCAIP, CRH, **CATSPER3**, PRKCI, **NDUFAF2**, **LRRK2**, **SNCA**, **SYT17**, **STK39**, **BAG3**, PARD6B, **PARD3**, UNC13B, **GPNMB**, **TRIM40**, **CRHR1**, **RIMS1**, LDLR, STX10, CLTC
Intracellular transport	20	1390	0.61	8.84 × 10^−5^	RAB3A, **STBD1**, **APOE**, STX6, KPNB1, PRKCI, **GAK**, **SNCA**, **KPNA1**, **SYT17**, STX16, **PARD3**, **DLG2**, UNC13B, VTI1A, **SNX13**, **RIMS1**, LDLR, STX10, CLTC
Regulation of neurotransmitter secretion	6	55	1.49	8.84 × 10^−5^	RAB3A, SNCAIP, **LRRK2**, **SNCA**, UNC13B, **RIMS1**
Regulation of neurotransmitter transport	7	92	1.33	8.84 × 10^−5^	RAB3A, SNCAIP, CRH, **LRRK2**, **SNCA**, UNC13B, **RIMS1**
Regulation of cellular localization	15	766	0.74	8.84 × 10^−5^	PARD6A, RAB3A, STX6, SNCAIP, CRH, PRKCI, **NDUFAF2**, **LRRK2**, **SNCA**, **BAG3**, **PARD3**, UNC13B, **TRIM40**, **RIMS1**, CLTC
Membrane organization	15	729	0.77	8.84 × 10^−5^	APOB, **VAMP4**, **APOE**, STX6, **TMEM175**, PRKCI, **GAK**, **SNCA**, **SYT17**, STX16, VTI1A, **RIMS1**, LDLR, STX10, CLTC
Membrane fusion	8	170	1.13	8.84 × 10^−5^	**VAMP4**, STX6, **TMEM175**, **SYT17**, STX16, VTI1A, **RIMS1**, STX10
Golgi ribbon formation	4	11	2.01	8.84 × 10^−5^	**VAMP4**, STX6, STX16, VTI1A
Regulation of synaptic vesicle cycle	5	46	1.49	0.00016	RAB3A, **LRRK2**, **SNCA**, UNC13B, **RIMS1**
Golgi organization	6	95	1.25	0.00022	**VAMP4**, STX6, **LRRK2**, **GAK**, STX16, VTI1A
Chemical synaptic transmission	10	402	0.85	0.00024	RAB3A, **APOE**, CRH, **LRRK2**, **SNCA**, **SYT17**, **DLG2**, UNC13B, **RIMS1**, **GPR19**
Vesicle organization	9	318	0.9	0.00024	RAB3A, **VAMP4**, STX6, **TMEM175**, PRKCI, **SYT17**, STX16, VTI1A, STX10
Synaptic vesicle cycle	6	100	1.23	0.00024	RAB3A, **GAK**, **SNCA**, **SYT17**, UNC13B, **RIMS1**
Regulation of synaptic vesicle exocytosis	4	24	1.67	0.00028	RAB3A, **LRRK2**, UNC13B, **RIMS1**
Cell-cell signaling	15	1073	0.6	0.00042	PARD6A, RAB3A, **WNT3**, **APOE**, CRH, **LRRK2**, **SNCA**, **SYT17**, **DLG2**, UNC13B, **GPNMB**, **CRHR1**, **RIMS1**, **GPR19**, CLTC
Regulation of synaptic vesicle transport	4	28	1.61	0.00044	RAB3A, **LRRK2**, UNC13B, **RIMS1**
Regulation of calcium ion-dependent exocytosis	5	66	1.33	0.00045	RAB3A, **LRRK2**, **SYT17**, UNC13B, **RIMS1**
Establishment of localization	32	4248	0.33	0.00045	RAB3A, APOB, **VAMP4**, **STBD1**, **APOE**, STX6, **TMEM175**, **CATSPER3**, KPNB1, PRKCI, **LRRK2**, **GAK**, **IP6K2**, **SNCA**, **KPNA1**, **CTSB**, **SYT17**, STX16, **SLC44A1**, **PARD3**, **DLG2**, **ITGA8**, UNC13B, VTI1A, **CRHR1**, NPNT, **SNX13**, **RIMS1**, **FAM49B**, LDLR, STX10, CLTC
Chylomicron remnant clearance	3	8	2.03	0.00057	APOB, **APOE**, LDLR
Bicellular tight junction assembly	4	32	1.55	0.0006	PARD6A, PRKCI, PARD6B, **PARD3**
Cytosolic transport	6	132	1.11	0.00065	STX6, **GAK**, STX16, VTI1A, STX10, CLTC
Protein localization	20	1966	0.46	0.00071	RAB3A, APOB, **APOE**, STX6, **BIN3**, KPNB1, PRKCI, **LRRK2**, **GAK**, **KPNA1**, STX16, **PARD3**, **DLG2**, **ITGA8**, VTI1A, NPNT, **SNX13**, **RIMS1**, STX10, CLTC
Regulation of neurotransmitter levels	8	295	0.89	0.00073	RAB3A, SNCAIP, **LRRK2**, **SNCA**, **SYT17**, **SLC44A1**, UNC13B, **RIMS1**
Cellular component assembly	22	2343	0.43	0.00073	PARD6A, APOB, **VAMP4**, **APOE**, **BRIP1**, **TMEM175**, **MCCC1**, **BIN3**, KPNB1, PRKCI, **NDUFAF2**, **LRRK2**, **GAK**, MCCC2, **THSD4**, **SCAF11**, PARD6B, **PARD3**, UNC13B, **PET117**, **RIMS1**, CLTC
Retrograde transport, endosome to Golgi	5	79	1.25	0.00073	STX6, STX16, VTI1A, STX10, CLTC
Establishment of protein localization	17	1467	0.52	0.00073	RAB3A, APOB, **APOE**, STX6, KPNB1, PRKCI, **KPNA1**, STX16, **PARD3**, **DLG2**, **ITGA8**, VTI1A, NPNT, **SNX13**, **RIMS1**, STX10, CLTC
Cellular localization	21	2180	0.44	0.0008	RAB3A, **STBD1**, **APOE**, STX6, KPNB1, PRKCI, **LRRK2**, **GAK**, **SNCA**, **KPNA1**, **SYT17**, STX16, **PARD3**, **DLG2**, UNC13B, VTI1A, **SNX13**, **RIMS1**, LDLR, STX10, CLTC

*The protein products of genes with significant associations are highlighted in bold*.

Several genes occupy central nodes in the protein network: *APOE* occupies a central node in the protein network and in our analysis is associated with the family history of dementia. *APOE* is a well-established AD risk factor (62) with an important role in normal brain function (63) and the *APOE* e4 allele has been associated with cognitive decline in PD (10, 64, 65). *LRRK2* is also occupying a central node in the protein network: *LRRK2* has a dual role as a PD risk factor and a gene involved in PD pathogenesis (66, 67) and encodes a protein kinase involved in autophagy. *SNCA* also occupies a central node in the PPI and is a key player in PD pathogenesis (68).

Taken together with the results of the association analyses, these results are consistent with the hypothesis that genetic variation that affects the functioning of protein-protein interaction networks can contribute to the differential presentation of PD symptoms. In addition, it is important to note that a number of these genes are members of known networks and hubs, whereas others are not.

## Discussion

Here we present the results of association analyses of baseline clinical features in PD with genetic variants that have been shown to be significant in prior case-control GWAS to confer PD risk or have been identified as PD-associated genes in the MDSgene database. We analyzed discrete clinical phenotypic features and test scores in a two-pronged approach: in the first, we evaluated their association with individual common SNPs that have been demonstrated in case-control GWAS to confer PD-risk; in the second we used gene-level tests to evaluate the association of these phenotypic features with low frequency (1–5% MAF) and rare (<1% MAF) variants in both pathogenic PD genes and the genes conferring PD-risk identified by case-control GWAS. The rationale of this approach is based on the hypothesis that individual discrete phenotypic characteristics may be differentially affected by the action of individual SNPs that tag a particular PD-risk haplotype, and/or multiple variants at a particular gene. Furthermore, the observed associations may reflect the effects of variants with different MAF. The alternative to this hypothesis is that the single SNPs and variants within a gene that confer PD-risk affect groups of clinical features or test scores more uniformly.

Our results support this hypothesis: individual common SNPs conferring PD risk are associated with phenotypic traits mostly in a non-overlapping manner, and gene-level tests reveal associations with individual clinical features and test scores that are often differentially affected, though at times have overlapping effects. This raises the intriguing possibility that individual phenotypic characteristics of a neurodegenerative disease such as PD that are associated with a specific gene may be related with the same phenotypic characteristic in a different neurodegenerative disease/syndrome. This may allow for the development of a “polyphenic” risk score to complement polygenic composite risk scores that already have been developed for Alzheimer's disease and other diseases (69).

It is interesting to point out certain associations that may hint to pathogenetic links between PD and other disorders. The relationship between PD and ET has long been a matter of debate (70). In our cohort, gene-based tests reveal an association between LRRK2 and history of essential tremor. This finding suggests that genetic variation at *LRRK2* may provide a link between long-standing ET and the development of PD at least in some cohorts. The presence of neuropathy in our cohorts is associated with variants in the *ELOVL7* and *NDUFAF2* genes that are located in the same region on chromosome 5. Clinically, peripheral neuropathy has been reported in PD, however, its cause remains unclear, potentially reflecting medication adverse effects (71).

Another striking association in our cohort is that of *SNCA* with cognitive impairment. The role of common variants at *SNCA* as PD risk factors, as well as rare gene variants as pathogenic mutations has been clearly demonstrated over the last two decades. Our findings suggest that multiple, less common variants at SNCA, not necessarily pathogenic variants, may affect cognition in PD patients.

The reported prevalence and incidence estimates in PD show a 1.5:1 male to female ratio (72). Here we find that sex often differentially affects an association with a particular phenotypic trait, either in the form of a symptom or a test score: some of the associations are significant for males or females, whereas others in both sexes. This suggests that sex may have a differential effect on the phenotypic manifestation of genetic PD risk.

As would be expected from our current understanding of the genetic mechanisms underlying PD, protein-protein interaction network analysis demonstrates that about two-thirds of the genes with significant associations are members of previously identified networks. However, about a third of the genes appear unconnected to these networks. This raises the interesting possibility that as yet unidentified gene networks and connections may be implicated in phenotypic manifestations, in either a deleterious or protective role.

It is important to stress that the analyses presented here are based on patient-reported initial symptoms and symptoms at baseline encounter, as well as objective test scores determined at the baseline encounter. Longitudinal evaluation of this and other cohorts through a standardized assessment at annual intervals will enable the extension of this analysis to determine whether the impact of the genotypes on the clinical phenotype and test scores is among other factors dependent on disease subtype, severity and duration. It also will be informative to undertake additional analyses that cluster individual symptoms and analyze their associations with genetic risk factors.

One limitation to our study is the inclusion of both *de novo* and previously diagnosed patients. Therefore, our cohort is likely more heterogeneous than an exclusively *de novo* cohort such as the PPMI cohort. However, given that the study participation originates in a community-based cohort, it is likely more representative of the phenotypic spectrum that is typically observed in clinician practices. Furthermore, the PD diagnosis in our cohort according to published diagnostic criteria (21) is ascertained at the baseline visit and can also be reliably ascertained at annual intervals using the EMR-based SCDS, thus providing high clinical diagnostic accuracy. In addition, the use of SCDS allows for detailed and accurate clinical data collection in a routine clinical practice, thus more accurately reflecting the clinical course.

A second limitation of this study is that the sample size of our cohort limits its power to detect associations. While none of the associations with common PD-risk SNPs reach genome-wide significance (~5 × 10^−8^) (Tables 1, 2, Supplementary Tables 4, 5), gene-level tests using rare variants identify four associations with baseline clinical features that approach or reach significance for the number of mapped genes (2.81 × 10^−6^): *TOX3* and *SULT1C2* with UPDRS IV-total score, *GPNMB* with bradykinesia, *CATSPER3* (73) with anxiety (Table 3, Supplementary Table 6). It is important to point out in this context that the genes included in this analysis have been previously clearly associated with PD-risk in case-control GWAS. Nevertheless, given the size of our cohort, it will be informative to evaluate the reproducibility of our findings in other cohorts.

In summary, our analysis shows that common SNPs conferring PD-risk, as well as low-frequency and rare variants in genes implicated in PD/parkinsonism are associated with distinct phenotypic characteristics at baseline presentation in our PD cohorts, supporting the hypothesis that the genetic background significantly affects disease presentation and raising the possibility that it also affects disease course and severity. The associations observed are often, but not always, dependent on sex. It is conceivable that this is related to the observed PD prevalence and incidence estimates that point to PD-risk differences based on sex. Finally, this analysis identifies different patterns in protein interaction networks that may underlie disease phenotype and pathogenesis. Longitudinal studies of this and other PD cohorts using this approach can provide insights on the impact of genetic risk factors on disease severity and progression, and enhance our understanding of the underlying pathogenetic mechanisms contributing to PD.

## Data Availability Statement

The datasets presented in this article are not readily available because this would jeopardize patient confidentiality. Additional information can be made available to qualified researchers after completing a material transfer agreement that maintains patient confidentiality with NorthShore University HealthSystem. Requests to access the datasets should be directed to the corresponding author.

## Ethics Statement

The studies involving human participants were reviewed and approved by NorthsShore University HealthSystem Institutional Review Board. The patients/participants provided their written informed consent to participate in this study.

## Author Contributions

KM designed the study and wrote the manuscript. BC performed data analysis and contributed to the writing. KM, DM, and RF designed clinical instruments used in the study. KM, DM, APP, BS, and NK provided the clinical assessment. AP, LG, and RV provided research assistance. JW, AE, and HY processed genomic and clinical data. KM, BC, RF, and DM edited the manuscript. All authors contributed to the article and approved the submitted version.

## Conflict of Interest

The authors declare that the research was conducted in the absence of any commercial or financial relationships that could be construed as a potential conflict of interest.
