# Supplementary material for: Variable Effects of PD-Risk Associated SNPs and Variants in Parkinsonism-Associated Genes on Disease Phenotype in a Community-Based Cohort
Source: Front Neurol. 2021 Apr 14;12:662278. doi: 10.3389/fneur.2021.662278 (PMC8079937; doi:10.3389/fneur.2021.662278)
Supplement: Supplementary file 2 [file Table_2.DOCX]

**Supplemental Table 4**: Significant associations of binomial traits with individual common PD-risk SNPs, not including years-from-diagnosis as a covariate

| **Clinical Feature** | **SNP** | **Nearest Gene** | **Distance (kb)** | **MAF this study** | **Sex** | **Model** | **unadjusted *p* value** | **Bonferroni corrected *p* value** | **OR** | **95% CI** |
| --- | --- | --- | --- | --- | --- | --- | --- | --- | --- | --- |
| Family history of dementia n≥2 | rs429358  APOE ε4 dose | *APOE* | 0 | 12.4  11.1 | Both  Females  Both | Additive  Additive  Additive | 2.79 × 10^-4^  7.05 × 10^‑5^  3.05 × 10^-4^ | 0.03855  0.00972  0.04208 | 4.13  8.98  4.07 | 1.92-8.87  3.04-26.5  1.90-8.72 |
| Presence of neuropathy | rs186798 | *ELOVL7* | 0 | 13.5 | Females | Additive | 2.97 × 10^-5^ | 0.00409 | 3.85 | 2.04-7.24 |
| History of essential tremor | rs12528068 | *RIMS1* | 108.6 | 28.6 | Males | Additive | 3.22 × 10^-4^ | 0.0445 | 2.09 | 1.40-3.12 |
| ***Initial motor symptom*** | | | | | | | | | | |
| Bradykinesia-Reduced arm swing | rs34311866 | *TMEM175* | 0.5 | 25.3 | Both | Additive | 1.69 × 10^-4^ | 0.0231 | 2.30 | 1.49-3.54 |
| ***Initial non-motor symptom*** | | | | | | | | | | |
| Depression | rs6116989 | *BRIP1* | 0 | 14.9 | Males | Additive | 3.61 × 10^-4^ | 0.4232 | 2.58 | 1.54-4.31 |
| ***Disease subtype*** | | | | | | | | | | |
| Tremor-predominant subtype | rs9468199 | *LOC100507172* | 3.2 | 17.4 | Both | Additive | 3.04 × 10^-4^ | 0.0420 | 2.16 | 1.42-3.28 |
| ***Non-motor symptoms at baseline*** | | | | | | | | | | |
| Cognitive impairment | rs4653767 | *ITPKB* | 9.9 | 26.6 | Both | Additive | 2.74 × 10^-4^ | 0.03775 | 1.97 | 1.37-2.84 |
| Orthostatism | rs34025766 | *LCORL* | 0 | 17.0 | Males | Additive | 8.21 × 10^-5^ | 0.01333 | 3.04 | 1.75-5.29 |
| Hallucinations | rs55961674 | *KPNA1* | 0 | 19.9 | Males | Additive | 2.82 × 10^-4^ | 0.03807 | 2.71 | 1.58-4.64 |

**Supplemental Table 5.** Significant associations of test scores with common PD-risk SNPs, not including years-from-diagnosis as a covariate

| **Clinical Feature** | ***SNP*** | **Nearest Gene** | **Distance to nearest gene (kb)** | **MAF this study** | **Sex** | **Model** | **Unadjusted *p* value** | **Bonferroni corrected *p* value** | **β** | **95% CI** |
| --- | --- | --- | --- | --- | --- | --- | --- | --- | --- | --- |
| ESS score | rs7134559 | *SCAF11* | 33.2 | 43.0 | Females | Additive | 3.00 × 10^-4^ | 0.0414 | 0.323 | 0.151-0.496 |
| MMSE score | rs823118 | *NUCKS1* | 4.1 | 42.1 | Males | Additive | 3.20 × 10^-4^ | 0.0441 | 0.245 | 0.112-0.377 |
| H&Y stage | rs12813102 | *GPR19* | 0 | 4.2 | Both  Males | Additive  Additive | 2.89 × 10^-4^  8.49 × 10^-5^ | 0.0399  0.0117 | 1.68  1.71 | 0.777-2.59  0.862-2.55 |
| UPDRS IV-Dyskinesias | rs12528068 | *RIMS1* | 108.6 | 28.6 | Females | Additive | 2.77 × 10^-4^ | 0.0382 | 0.472 | 0.221-0.724 |
| UPDRS-IV-Other | rs224750 | *PARD3* | 167.5 | 42.1 | Females | Additive | 2.44 × 10^-4^ | 0.0337 | 0.333 | 0.158-0.509 |

CI=confidence interval; ESS = Epworth Sleepiness Scale; MMSE= Mini Mental Status Examination; H&Y= Hoehn & Yahr stage; OR= odds ratio; SNP=single nucleotide polymorphism; UPDRS=Unified Parkinson’s Disease Rating Scale

**Supplemental Table 6.** Significant Associations in Gene-Based Sequence Kernel Association Tests, excluding covariates

| **Clinical Feature** | **Analysis N** | **N with condition in this study (%)** | **Variant MAF Criteria** | **Gene** | **Number of Variants** | **Unadjusted *p* (permutation *p*)** | **Bonferroni Corrected *p*** |
| --- | --- | --- | --- | --- | --- | --- | --- |
| ***Clinical history*** | | | | | | | |
| Essential tremor | 779 | 144 (18.3) | ≤ 5% | *LRRK2* | 37 | 3.54 × 10^-5^ (0) | 0.0041 |
| ***Test scores*** | | | | | | | |
| MMSE | 783 | - | ≤ 1% | *FAM171A1* | 15 | 0.000337 (0.0097) | 0.0357 |
| UPDRS III | 743 | - | ≤ 5%  ≤ 1% | *NUCKS1*  *NUCKS1* | 11  8 | 0.000355 (0.0001)  0.000192 (0.0005) | 0.0416  0.0204 |
| UPDRS -I | 768 | - | ≤ 1% | *TMEM163* | 19 | 0.000245 (0.0018) | 0.0260 |
| UPDRS IV- dyskinesia subscore | 786 | - | ≤ 1%  ≤ 1% | *SULT1C2*  *TOX3* | 6  16 | 7.26 × 10^-5^ (0.004)  0.000177 (0.0067) | 0.00770  0.0188 |
| UPDRS IV-fluctuation subscore | 786 | - | ≤ 5%  ≤ 1%  ≤ 1%  ≤ 1% | *BAG3*  *MCCC1*  *TOX3*  *SULT1C2* | 6  14  16  6 | 0.000466 (0.0029)  4.28 × 10^-5^ (0.0012)  0.000197 (0.0035)  0.000264 (0.0036) | 0.0428  0.00454  0.0209  0.028 |
| UPDRS IV - other subscore | 786 | - | ≤ 5%  ≤ 1%  ≤ 1% | *STBD1*  *TOX3*  *STBD1* | 2  16  2 | 0.000406 (0.0022)  0.000198 (0.0014)  0.000406 (0.0025) | 0.0475  0.0210  0.0431 |
| UPDRS IV-total score | 786 | - | ≤ 1%  ≤ 1% | *TOX3*  *SULT1C2* | 16  6 | 7.92 × 10^-7^ (0.0001)  5.65 × 10^-6^ (0.0005) | 8.37 × 10^-5^  5.99 × 10^-4^ |
| UPDRS-VI | 773 | - | ≤ 1% | *TRIM40* | 2 | 1.05 × 10^-5^ (0.00115) | 0.00111 |
| Dyskinesia at baseline: chorea | 675 | 61 (7.7) | ≤ 5% | *GPR19* | 3 | 0.000131 (0.0016) | 0.0154 |
| Dyskinesia severity at baseline: severe | 784 | 61 (7.7) | ≤ 5% | *GPR19* | 3 | 8.84 × 10^-5^ (0.001) | 0.0103 |
| ***Initial motor symptoms*** | | | | | | | |
| Bradykinesia (reduced arm swing) | 786 | 45 (5.7) | ≤ 1% | *IP6K2* | 3 | 0.000196 (0.0083) | 0.0208 |
| Bradykinesia (generalized) | 786 | 65 (8.2) | ≤ 1% | *SCAF11* | 15 | 0.00037 (0.0289) | 0.0392 |
| Postural tremor | 786 | 140 (17.8) | ≤ 1% | *FAM49B* | 15 | 2.14 × 10^-5^ (0.0001) | 0.00227 |
| **Motor symptoms at baseline** | | | | | | | |
| Bradykinesia | 786 | 779 (98.9) | ≤ 5%  ≤ 5%  ≤ 1%  ≤ 1% | *GPNMB*  *THSD4*  *GPNMB*  *DNAH17* | 20  133  17  61 | 4.41 × 10^-5^ (0.0398)  0.000116 (0.006)  3.30 × 10^-6^ (0.0190)  0.000291 (0.0248) | 0.00516  0.0135  0.000350  0.0308 |
| ***Initial non-motor symptom*** | | | | | | | |
| Depression | 786 | 125 (15.9) | ≤ 1% | *ITGA8* | 26 | 0.000306 (0.0014) | 0.0324 |
| ***Non-motor symptoms at baseline*** | | | | | | | |
| Cognitive impairment | 786 | 106 (13.5) | ≤ 5% | *SNCA* | 19 | 4.15 × 10^-4^ (0.0002) | 0.00486 |
| Constipation | 786 | 157 (19.9) | ≤ 1% | *ITGA8* | 26 | 4.19 × 10^-5^ (0.0001) | 0.00444 |
| Orthostatism | 786 | 56 (7.1) | ≤ 5% | *PET117* | 4 | 0.000263 (0.0219) | 0.0307 |
| UPDRS IV-Orthostasis | 786 | 62 (7.9) | ≤ 5%  ≤ 1% | *STBD1*  *STBD1* | 2  2 | 4.93 × 10^-5^ (0.0033)  4.93 × 10^-5^ (0.0037) | 0.00577  0.00523 |
| Hallucinations | 786 | 49 (6.2) | ≤ 5% | *LRRK2* | 36 | 1.73 × 10^-5^ (0.001) | 0.0202 |
| Dysphagia | 786 | 47 (6.0) | ≤ 1% | *FAM49B* | 15 | 0.000183 (0.0049) | 0.0194 |
| Anxiety | 786 | 50 (6.3) | ≤ 1% | *CATSPER3* | 7 | 5.31 × 10^-8^ (0.0008) | 5.62 × 10^-6^ |
| ***Family history*** | | | | | | | |
| Dementia (1 family member) | 786 | 160 (20.3) | ≤ 1% | *SLC44A1* | 16 | 0.00015 (0.0004) | 0.0159 |
| Dementia (≥ 2 family members) | 786 | 24 (3.0) | ≤ 1% | *ASXL3* | 27 | 2.21 × 10^-5^ (0.0033) | 0.00234 |
| Dementia (≥ 1 family member) | 786 | 184 (23.4) | ≤ 1% | *ASXL3* | 27 | 4.51 × 10^-5^ (0) | 0.00478 |
